# Improvement in asymmetric dimethylarginine and oxidative stress in patients with limb salvage after autologous mononuclear stem cell application for critical limb ischemia

**DOI:** 10.1186/s13287-017-0622-2

**Published:** 2017-07-12

**Authors:** Juraj Madaric, Martina Valachovicova, Ludovit Paulis, Jana Pribojova, Renata Mateova, Katarina Sebekova, Luba Postulkova, Terezia Madaricova, Maria Bucova, Martin Mistrik, Ivan Vulev

**Affiliations:** 1 National Institute of Cardiovascular Diseases, Slovak Medical University, Bratislava, Slovakia; 2 Slovak Medical University, Bratislava, Slovakia; 3 Institute of Molecular BioMedicine, Faculty of Medicine Comenius University, Bratislava, Slovakia; 4 Institute of Imunology, Faculty of Medicine Comenius University, Bratislava, Slovakia; 5 Clinic of Haematology and Transfusiology, Faculty Hospital, Bratislava, Slovakia

**Keywords:** Cell therapy, Oxidative stress, Asymmetric dimethylarginine, Angiogenesis, Critical limb ischemia

## Abstract

**Background:**

Asymmetric dimethylarginine (ADMA), an endogenous inhibitor of nitric oxide synthase, acts as an inhibitor of angiogenesis and is associated with an increased risk of cardiovascular mortality. Administration of stem cells may affect endogenous mechanisms that regulate ADMA production and metabolism. The aim of the present study was to analyze ADMA concentration and changes in oxidative stress in patients with advanced critical limb ischemia (CLI) after bone marrow-derived mononuclear cell (BM-MNC) therapy.

**Methods:**

Fifty patients (age 64 ± 11 years, 44 males, 6 females) with advanced CLI (Rutherford category 5 or 6) not eligible for revascularization were treated by intramuscular (*n* = 25) or intra-arterial (*n* = 25) injection of 40 ml BM-MNC concentrate. Patients with limb salvage and improved wound healing after 6 months were considered responders to cell therapy. The concentrations of markers of oxidative stress and angiogenesis were analyzed before, and at 3 and 6 months after BM-MNC delivery.

**Results:**

At 6-month follow-up, four patients died of reasons unrelated to stem cell therapy. Among the survivors, 80% (37/46) showed limb salvage and improved wound healing. At 6 months follow-up, ADMA concentration significantly decreased in patients with limb salvage (1.74 ± 0.66 to 0.90 ± 0.49 μmol/L, *p* < 0.001), in parallel with decreased tumor necrosis factor (TNF)-α (2.22 ± 0.16 to 1.94 ± 0.38 pg/ml, *p* < 0.001), and increased reduced glutathione (6.96 ± 3.1 to 8.67 ± 4.2 μmol/L, *p* = 0.02), superoxide dismutase activity (168 ± 50 to 218 ± 37 U/L, *p* = 0.002), and coenzyme Q10 concentration (468 ± 182 to 598 ± 283 μg/L, *p* = 0.02). The number of delivered BM-MNCs significantly correlated with the decrease in ADMA concentration at 3 months (*p* = 0.004, *r* = −0.48) and the decrease in TNF-α concentration at 6 months (*p* = 0.03, *r* = −0.44) after cell delivery. ADMA or TNF-α improvement did not correlate with the number of applied CD34^+^ cells, C-reactive protein concentration, leukocyte count, or the dose of atorvastatin.

**Conclusions:**

The therapeutic benefit of BM-MNC therapy is associated with reduced ADMA levels and oxidative stress. Regulation of the ADMA-nitric oxide axis and improved antioxidant status may be involved in the beneficial effects of stem cell therapy.

**Trial registration:**

The study was approved and retrospectively registered by ISRCTN registry, ISRCTN16096154. Registered on 26 July 2016.

## Background

**Fig. 1:**
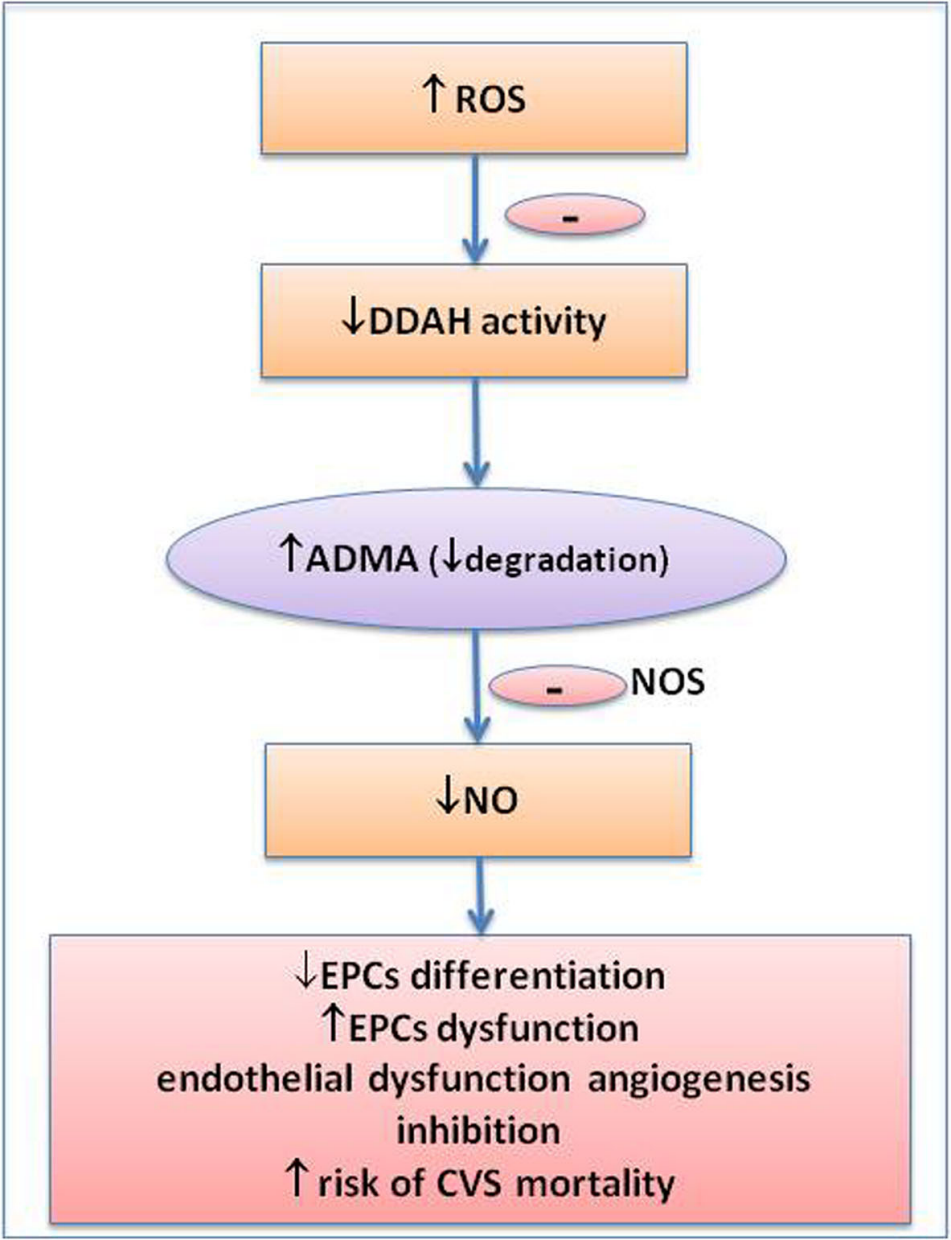
Oxidative stress and stem cell dysfunction. *ADMA* asymmetric dimethylarginine, *CVS* cardiovascular, *DDAH* dimethylarginine dimethylaminohydrolase, *EPC* endothelial progenitor cell, *NO* nitric oxide, *NOS* endothelial nitric oxide synthase, *ROS* reactive oxygen species

Asymmetric dimethylarginine (ADMA), an endogenous inhibitor of nitric oxide synthase (NOS), regulates the rate of nitric oxide (NO) formation, acts as an inhibitor of angiogenesis, and is associated with an increased risk of cardiovascular morbidity and mortality [1–4]. Increased ADMA plasma concentrations predict major adverse cardiovascular events in patients with advanced peripheral artery disease [5]. Increased oxidative stress contributes to elevated ADMA. In turn, increased serum ADMA concentration is associated with increased vascular oxidative stress, as demonstrated by the upregulation of circulating markers of oxidative stress. The precise relationship between serum ADMA and oxidative stress remains unclear. The enzyme responsible for ADMA degradation, dimethylarginine dimethlyaminohydrolase (DDAH), is redox-sensitive as its activity decreases in the presence of increased oxidative stress. Therefore, oxidative stress increases ADMA synthesis and inhibits its degradation [6–8] (Fig. 1). Preclinical models indicate that antioxidant therapy improves neovascularization when given in combination with bone marrow-derived mononuclear cells (BM-MNCs) [9–11].

The aim of the present study was to analyze ADMA concentration and changes in oxidative stress in patients with advanced critical limb ischemia (CLI) in response to BM-MNC therapy.

## Methods

### Patients

Fifty patients (age 64 ± 11 years, 44 males, 6 females) with advanced CLI (Rutherford category 5 or 6) who were not eligible for revascularization were treated by intramuscular (IM) (*n* = 25) or intra-arterial (IA) (*n* = 25) injection of 40 ml BM-MNC concentrate. The mode of application was based on computerized randomization with equal sex and diabetes mellitus distribution in both groups. The etiology of arterial obliteration was atherosclerosis in 45 patients and thromboangiitis obliterans (Buerger disease) in 5 patients.

#### Inclusion criteria

Inclusion criteria were: 1) patients over 18 years of age with ischemic skin lesions (ulcer or gangrene) and Rutherford category 5 or 6 CLI according to the TASC classification (minor or major tissue loss) [12]; 2) CLI defined by ankle-brachial index ≤0.4, or ankle systolic pressure <50 mmHg, or toe systolic pressure <30 mmHg, and transcutaneous oxygen pressure (tcpO_2_) <30 mmHg; and 3) no option for endovascular or surgical revascularization as determined by both vascular surgeon and interventionalist.

#### Exclusion criteria

Exclusion criteria were: 1) life expectancy of <6 months; 2) evidence of malignancy during the previous 5 years; 3) critical coronary artery disease or unstable angina pectoris; 4) end-stage kidney disease; and 5) bone marrow disease, e.g., myelodysplastic syndrome, severe anemia, leukopenia, and thrombocytopenia.

### Method of BM-MNC isolation and administration

Under analgosedation with propofol, a total of 240 ml bone marrow was harvested from both posterior iliac crests. The bone marrow aspirate was processed with the SmartPreP2 Bone Marrow Aspirate Concentrate System (Harvest, Plymouth, MA, USA). The system uses gradient density centrifugation to provide 40 ml of bone marrow product rich for all blood elements within 15 min. The final product is defined by mix of cell types, including lymphocytoid cells, erythroblasts, monocytoid cells, and granulocytes. The mean nucleated cell count is about 100 × 10^6^/ml. Bone marrow leukocytes and platelets are concentrated five- to sevenfold, and red cells are depleted to a hematocrit of less than 5%. Up to 99% of cells are viable after centrifugation [13–15]. After centrifugation, the cell concentrate was administered either by IM application (under analgosedation with propofol by deep IM injection of approximately 1 ml into the muscles of the affected limb along the crural arteries), or by IA infusion (injection of 40 ml of cell concentrate with a percutaneous femoral approach using a 4 F catheter at the site of arterial occlusion of the affected limb at a rate of 800 ml/h).

### Preprocedural assessment and follow-up

All patients were examined before, at 90 days, and at 6 months after BM-MNC delivery. Peripheral blood tests such as blood count and assessment of basal serological parameters, including C-reactive protein (CRP), were performed. Venous blood samples were collected for the determination of circulating markers of oxidative stress. The concentrations of ADMA (by Human, ADMA ELISA Kit; Sunlong Biotech Co. Ltd, Zhejiang, China), vascular endothelial growth factor (VEGF), tumor necrosis factor (TNF)-α, interleukin (IL)-6 (all by Quantikine ELISA; R&D Systems, Abingdon, UK), apolipoprotein A (Apo(a)), and oxidized low-density lipoproteins (oxLDL) were assessed using commercially available enzyme-linked immunosorbent assay (ELISA) kits (both supplied by antibodies-online Inc., Atlanta, USA). The levels of malondialdehyde (MDA), reduced glutathione (GSH), vitamin C, vitamin E, coenzyme Q10, and retinol were measured by high-performance liquid chromatography. Protein carbonyls (PC) and superoxide dismutase (SOD) activity were measured spectrophotometrically (reagents by Randox Laboratories Ltd., Crumlin, UK). DNA fragmentation was assessed using comet assays. The ferric reducing ability of plasma was evaluated by ferric reducing ability of plasma (FRAP) assay. The total antioxidant status (TAS; colorimetric kit by Randox Laboratories Ltd., Crumlin, UK) and total peroxide concentration of plasma (by OxyStat ELISA, Biomedica Medizinprodukte GmbH & CO KG, Vienna, Austria) were estimated.

In the bone marrow concentrate the total concentration of mononuclear cells and CD34^+^ cells was evaluated.

The following characteristics of limb ischemia were detected. Measurement of the resting ankle-brachial index (ABI) was performed according to validated standards [16]. TcpO_2_ of the affected limb was assessed using a TCM400 Mk2 monitor (Radiometer Medical ApS, Copenhagen, Denmark). TcpO_2_ was measured at the forefoot in the supine position with an electrode at a temperature of 44°C. Wound characteristics were documented by digital photography. Wound healing was evaluated by two independent physicians. The pain score was determined using a visual analog scale (VAS) and graded 0–10. Patients were discharged the day after the procedure on dual antiplatelet therapy (aspirin plus clopidogrel) and on statin therapy. All patients received conventional wound care during follow-up.

### Clinical outcomes

Patients with limb salvage and improvement in wound healing at 6 months were considered responders to cell therapy. Limb salvage was defined as the absence of amputation above the ankle joint. Other functional limb ischemia outcomes included in the evaluation were change in tcpO_2_, Rutherford category, and pain score after cell transplantation. The study was approved by the local Ethics Committee of the National Institute of Cardiovascular Diseases, Bratislava. All included patients were informed about the nature of the study and gave their written informed consent.

### Statistical analysis

Data evaluation was performed using the statistical software package SPSS v21.0 (SPSS Inc., Chicago, IL, USA). Discrete variables are presented as counts and percentages. Gaussian distributions of data were tested by the Kolmogorov-Smirnov test. Continuous variables are presented as the mean ± SD. For comparisons between the baseline and 6-month follow-up values, the paired Student *t* test was used for normally distributed values, Wilcoxon matched pairs test for nonparametric variables, and McNemar's test for categorical variables. For correlation analyses, Pearson’s *r* was calculated for normally distributed values and Spearman’s *r* was calculated for nonGaussian variables. For all analyses, a two-sided *p* value <0.05 was considered statistically significant.

## Results

The baseline characteristics of the patient population are listed in Table 1. During the 6 months of follow-up, four patients died of causes unrelated to stem cell application. In nine of the 46 surviving patients, major limb amputation was required because of CLI progression. At 6-month follow-up, 80% (37/46) of patients showed limb salvage and improved wound healing. The amputation-free survival endpoint was met in 74% (37/50) of patients, with no difference between IM versus IA application (72% vs. 76%, *p* = 1.0). Table 2 shows the parameters of limb ischemia in patients with limb salvage at 6 months. There was significant improvement in tcpO_2_, pain score, and CLI Rutherford category, whereas there was no difference in the ABI parameter.

**Table 1:** Baseline characteristics (*n* = 50)

Parameter	Baseline
Age (years)	64 ± 11
Male:female (*n*)	44:6
Diabetes mellitus	30 (60%)
Arterial hypertension	38 (76%)
Hyperlipidemia	27 (54%)
BMI (kg/m^2^)	28 ± 4
LVEF (%)	55 ± 7
Smoking	21 (42%)
Rutherford category	5.1 ± 0.3
Creatinine (μmol/L)	95 ± 51
CRP (mg/L)	22 ± 37
Leukocytes (10^9^/L)	9.1 ± 4.9
BM-MNCs in BMC (10^9^ cells)	4.2 ± 1.5
CD34^+^ in BMC (10^6^ cells)	28.1 ± 14.7
VEGF (pg/mL)	173 ± 191
Cholesterol (mmol/L)	4.3 ± 1.1
TAG (mmol/L)	1.4 ± 1.1
LDL (mmol/L)	2.8 ± 1.4
oxLDL (U/L)	24.3 ± 7.7
HDL (mmol/L)	1.2 ± 0.4
Atorvastatin (mg/day)	16 ± 11

*BMC* bone marrow concentrate, *BMI* body mass index, *BM-MNC* bone marrow-derived mononuclear cell, *CRP* C-reactive protein, *HDL* high-density lipoprotein, *LDL* low-density lipoprotein, *LVEF* left ventricle ejection fraction, *oxLDL* oxidized low-density lipoprotein, *TAG* triglycerides, *VEGF* vascular endothelial growth factor

**Table 2:** Functional limb ischemia outcomes in patients with limb salvage after BM-MNC application (*n* = 37)

	Baseline			6-month follow-up			*p* (baseline vs 6-month follow-up)		
	All patients (*n* = 37)	IM (*n* = 18)	IA (*n* = 19)	All patients (*n* = 37)	IM (*n* = 18)	IA (*n* = 19)	All patients	IM	IA
tcpO_2_ (mmHg)	16 ± 10	16 ± 10	17 ± 10	29 ± 14	30 ± 16	28 ± 12	<0.001	<0.001	<0.005
ABI	0.8 ± 0.3	0.8 ± 0.3	0.8 ± 0.3	0.9 ± 0.3	0.9 ± 0.2	0.9 ± 0.3	0.1	0.06	0.36
Pain scale (0–10)	4.4 ± 2.4	3.8 ± 2.1	5.0 ± 2.5	1.6 ± 1.6	1.2 ± 1.0	1.9 ± 1.9	<0.001	<0.001	<0.001
Rutherford category	5.0	5.0	5.0	4.0 ± 1.3	3.8 ± 1.3	4.2 ± 1.1	<0.001	<0.001	<0.005

*ABI* ankle-brachial index, *BM-MNC* bone marrow-derived mononuclear cell, *IA* intra-arterial, *IM* intramuscular, *tcpO*_*2*_ transcutaneous oxygen pressure

**Fig. 2:**
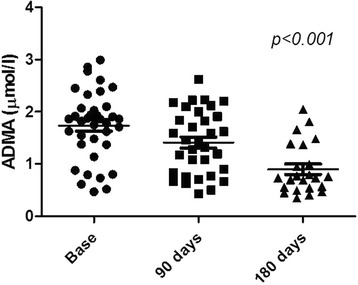
Decrease in ADMA concentration in patients with limb salvage after BM-MNC application. *p* < 0.001, 180 days vs. baseline. *ADMA* asymmetric dimethylarginine

Patients with limb salvage after cell therapy showed a significant decrease in ADMA concentration after 6 months (1.74 ± 0.66 to 0.90 ± 0.49 μmol/l, *p* < 0.001) and a significant decrease in TNF-α (2.22 ± 0.16 to 1.94 ± 0.38 pg/ml, *p* < 0.001) (Fig. 2 and Table 3).

**Table 3:** Decrease in ADMA, TNF-α, and inflammatory and related parameters in patients with limb salvage after BM-MNC application (*n* = 37)

	Baseline	6-month follow-up	*p*
ADMA (μmol/L)	1.74 ± 0.66	0.90 ± 0.49	<0.001
TNFα (pg/mL)	2.22 ± 0.16	1.94 ± 0.38	<0.001
Homocysteine (μmol/L)	12.2 ± 3.5	12.0 ± 3.1	0.29
CRP (mg/L)	14.3 ± 23.9	8.4 ± 13.7	0.15
IL-6 (pg/ml)	1.74 ± 0.9	1.76 ± 0.7	0.49
Leukocytes (10^9^/L)	8.6 ± 5.2	7.9 ± 2.3	0.20
Creatinine (μmol/L)	90 ± 38	89 ± 39	0.48

*ADMA* asymmetric dimethylarginine, *BM-MNC* bone marrow-derived mononuclear cell, *CRP* C-reactive protein, *IL* interleukin, *TNF* tumor necrosis factor

Table 4 shows biomarkers of oxidative stress in patients with limb salvage after cell therapy. There was a significant improvement in selected markers of antioxidant capacity (Apo(a), SOD, GSH, retinol, and coenzyme Q10), whereas improvements in other antioxidant parameters were borderline (vitamin C and vitamin C/vitamin E ratio). Serum levels of antioxidant markers FRAP and TAS remained unchanged at 6 months. Protein carbonyls decreased, whereas other markers of oxidative damage (MDA, oxLDL, and DNA fragmentation) showed no changes.

**Table 4:** Markers of oxidative stress in patients with limb salvage after BM-MNC application (*n* = 37)

Parameter of oxidative stress	Baseline	3-month follow-up	6-month follow-up	p*
Markers of endogenous antioxidant capacity				
SOD (U/L)	168 ± 50	178 ± 48	218 ± 37	0.002
GSH (μmol/L)	7.0 ± 3.1	8.8 ± 6.3	8.7 ± 4.2	0.02
Apo(a) (g/L)	0.26 ± 0.16	0.28 ± 0.17	0.33 ± 0.19	0.03
Retinol (μmol/L)	2.6 ± 0.9	2.4 ± 0.66	2.3 ± 0.7	0.01
Coenzyme Q10 (μg/L)	468 ± 182	594 ± 248	598 ± 283	0.02
Vitamin C (μmol/L)	22 ± 15	26 ± 16	29 ± 20	0.09
Vitamin C/Vitamin E	1.2 ± 0.9	1.4 ± 1	1.7 ± 1.4	0.07
FRAP (μmol/L)	951 ± 645	866 ± 235	845 ± 170	0.1
TAS (U/ml)	1.5 ± 0.3	1.5 ± 0.2	1.5 ± 0.2	0.2
Oxystat (μmol/L)	775 ± 563	885 ± 720	959 ± 796	0.1
Markers of oxidative stress damage				
PC (μmol/L)	131 ± 32	117 ± 30	106 ± 19	0.004
MDA (μmol/L)	1.1 ± 0.5	1.0 ± 0.6	1.1 ± 0.6	0.39
oxLDL (U/L)	23.4 ± 7.7	28.2 ± 17	25.9 ± 8.0	0.1
DNA fragmentation (AU)	59 ± 32	54 ± 22	54 ± 19	0.24

*Baseline versus 6-month follow-up

*Apo(a)* apolipoprotein A, *BM-MNC* bone marrow-derived mononuclear cell, *FRAP* ferric reducing ability of plasma, *GSH* reduced glutathione, *MDA* malondialdehyde, *oxLDL* oxidized low-density lipoproteins, *Oxystat* total peroxide concentration of plasma, *PC* protein carbonyls, *SOD* superoxide dismutase, *TAS* total antioxidant status

**Fig. 3:**
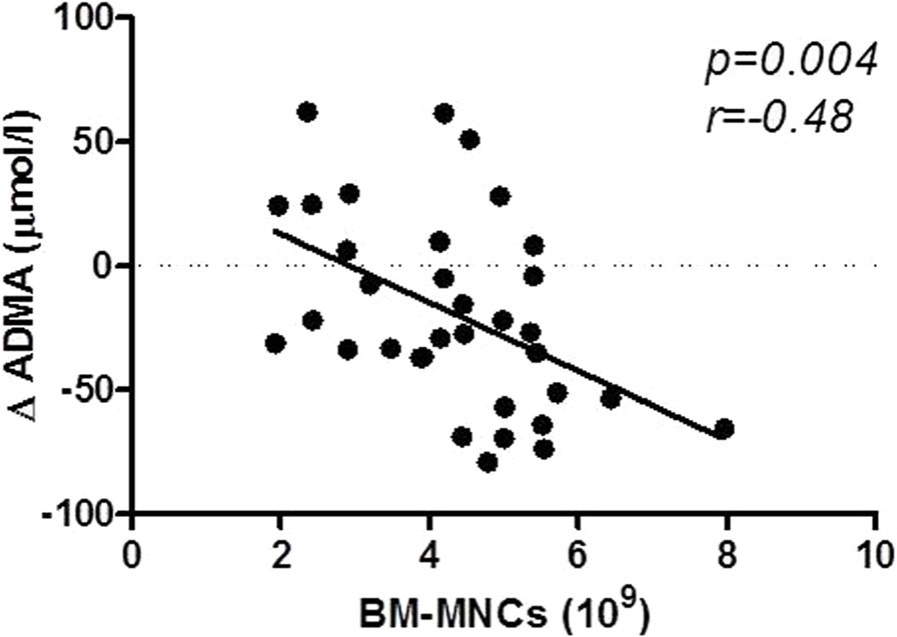
Decrease in ADMA concentration in relation to the number of delivered BM-MNCs at 3 months after cell delivery. *ADMA* asymmetric dimethylarginine, *BM-MNC* bone marrow-derived mononuclear cell

The serum concentration of ADMA before cell delivery correlated with VEGF levels (*p* < 0.001, *r* = 0.48). The dose of delivered BM-MNCs significantly correlated with the reduction in ADMA concentration at 3 months after cell application (*p* = 0.004, *r* = −0.48) (Fig. 3), TNF-α concentration at 6 months (*p* = 0.03, *r* = −0.44), and the decrease in peripheral leukocyte count at 3 months (*p* = 0.005, *r* = −0.52) and 6 months after cell delivery (*p* = 0.002, *r* = −0.51). There was no correlation between ADMA change and the number of applied CD34^+^ cells, CRP concentration, leukocyte count, or the dose of atorvastatin. No changes in homocysteine level, serum creatinine concentration, leukocytes, or IL-6 levels were observed between before and at 6 months after cell delivery (Table 3).

## Discussion

The present study investigated the effect of stem cell therapy on circulating ADMA levels and markers of oxidative stress in patients with CLI. The main findings can be summarized as follows: 1) BM-MNCs were associated with a significant reduction in serum ADMA levels, as well as TNF-α, and this was related to the dose of mononuclear cells; 2) BM-MNC therapy was associated with a reduction in the markers of oxidative stress. Taken together, these data indicate that regulation of the ADMA-NO axis and improvement of antioxidant balance may be involved in the beneficial effects of stem cell therapy in CLI patients.

Patients with limb salvage and improvement in wound healing at 6 months were defined as responders to cell therapy. Our mechanistic study is designed as a single arm mechanistic study without the control group. In addition, therapeutic benefit in terms of limb salvage and wound healing varies among various studies due to the number of modifying factors such as diabetes, renal failure, or extent of tissue loss. Nevertheless, our rates of therapeutic benefit (19.5% limb amputations) scores better as compared to the reported frequency of major limb amputation in a similar control, untreated population [17],

There was no difference between IA and IM application in all clinical parameters during follow-up. Likewise, there was no difference in all important measured parameters. Our results from a direct head-to-head comparison indicate that IM and IA methods of bone marrow cell delivery are effective for limb salvage and wound healing, with no significant differences in various functional surrogate endpoints between the techniques [18].

ADMA acts as an endogenous inhibitor of angiogenesis by impairing the NOS/NO pathway and NO bioavailability. High concentrations of ADMA are associated with low levels of endothelial progenitor cells (EPCs), suppressing their mobilization and differentiation [19]. Circulating EPCs are reduced and dysfunctional in various diseases in parallel with increased ADMA levels, such as diabetes, coronary heart disease, peripheral artery disease, and ischemic stroke. The reduction in ADMA levels might accelerate endothelial regeneration by promoting the mobilization of bone marrow-derived endothelial cells [20]. In our study, ADMA levels decreased proportionally to the dose of BM-MNCs suggesting a possible mechanistic link underlying the beneficial effect of cell therapy. ADMA is eliminated largely by the action of DDAH. As the activity of DDAH is typically reduced in conditions associated with oxidative stress, it is attractive to hypothesize that administration of BM-MNCs could modulate the ADMA concentration by increasing DDAH activity. This hypothesis warrants further mechanistic validation.

In addition, ADMA correlates with oxidative stress markers. Administration of ADMA significantly increases intracellular reactive oxygen species (ROS) production and reduces antioxidant capacity by decreasing SOD, catalase, and GSH activity [21]. On the other hand, oxidative stress increases ADMA synthesis and decreases its degradation. Corroborating these reports, our findings indicate a significant improvement in selected markers of antioxidant capacity or oxidative damage (Apo(a), SOD, GSH, retinol, coenzyme Q10, protein carbonyls) in parallel with a reduction in serum ADMA levels. Improvements in other antioxidant parameters were borderline or absent. Although improvements in selected parameters were more pronounced after IM application, it did not translate to a superior effect on surrogated or clinical outcomes.

TNF-α, an inflammatory cytokine involved in atherogenesis, is secreted from various cells including monocytes. ADMA increases the production of intracellular ROS and induces TNF-α expression. In our observations, the plasma levels of both ADMA and TNF-α decreased after cell application in correlation with the amount of cells administered and concomitant with the improvement of antioxidant status. Importantly, the decrease in ADMA levels did not correlate with parameters of inflammation, such as CRP, IL-6, or leukocyte concentration. Whether the application of BM-MNCs directly improved oxidative stress, with a subsequent positive influence on DDAH activity, ADMA concentration, and TNF-α, remains unclear from our data. Another possibility is that cell delivery decreased ADMA concentration, with further improvement in TNF-α activity and oxidative stress. Both mechanisms can be present simultaneously.

### Limitations

The main limitation of the study was the lack of a control group of “no-option” CLI patients who did not receive BM-MNCs. Another limitation was the relatively small number of treated patients. However, the current findings are hypothesis-generating for future larger, controlled trials. Additionally, evaluation of DDAH activity may be useful to clarify the important association between ADMA concentration and stem cell application.

## Conclusions

The goal of stem cell therapy in ischemic syndromes is to restore the vitality and functionality of ischemic tissues. Administration of BM-MNCs could positively affect tissue regeneration in CLI patients by decreasing ADMA concentration and attenuating oxidative stress. Regulation of the ADMA-NO axis and the ADMA synthesis pathway may represent a promising therapeutic target for stem cell therapy in regenerative interventions for cardiovascular diseases.

## Abbreviations

ABI: Ankle-brachial index
ADMA: Asymmetric dimethylarginine
Apo(a): Apolipoprotein A
BM-MNC: Bone marrow-derived mononuclear cell
CLI: Critical limb ischemia
CRP: C-reactive protein
DDAH: Dimethylarginine dimethylaminohydrolase
ELISA: Enzyme-linked immunosorbent assay
EPC: Endothelial progenitor cell
FRAP: Ferric reducing ability of plasma
GSH: Reduced glutathione
IA: Intra-arterial
IL: Interleukin
IM: Intramuscular
MDA: Malondialdehyde
NO: Nitric oxide
NOS: Nitric oxide synthase
oxLDL: Oxidized low-density lipoproteins
PC: protein carbonyls
ROS: Reactive oxygen species
SOD: Superoxide dismutase
TAS: Total antioxidant status
tcpO_2_: Transcutaneous oxygen pressure
TNF: Tumor necrosis factor
VEGF: Vascular endothelial growth factor
